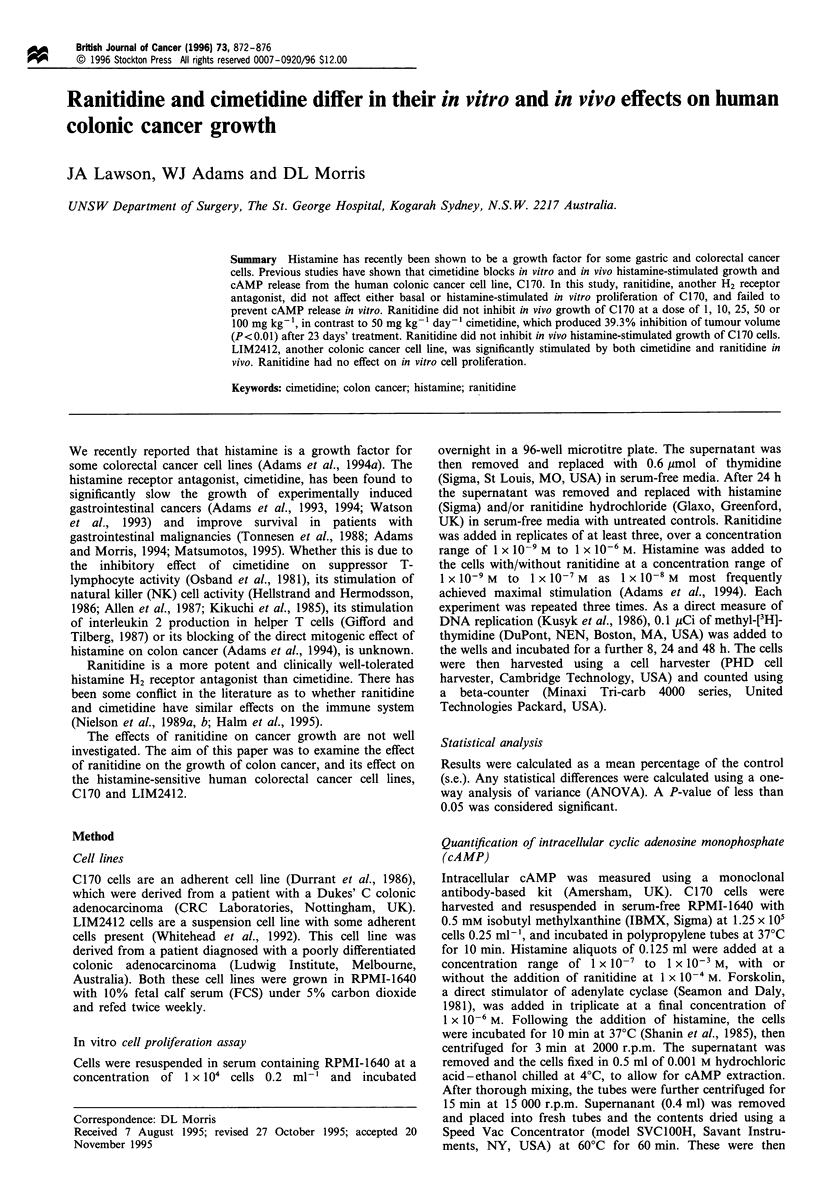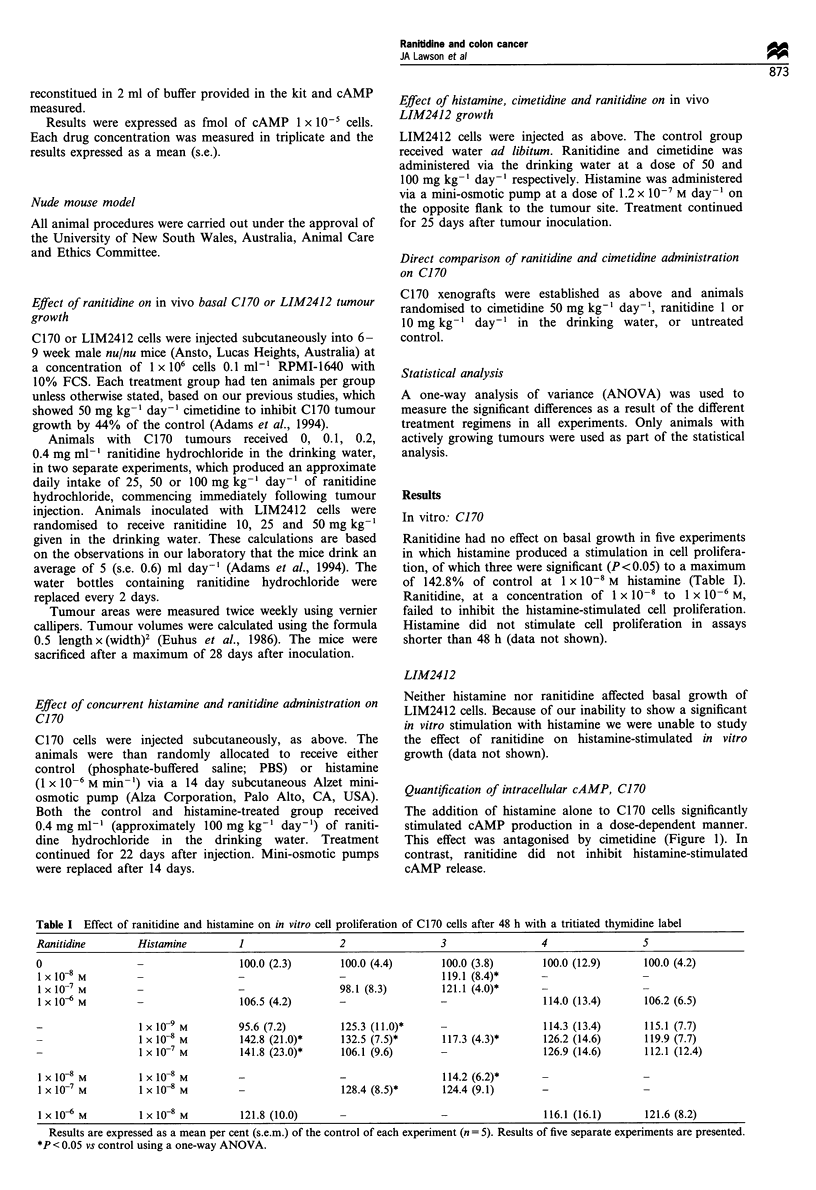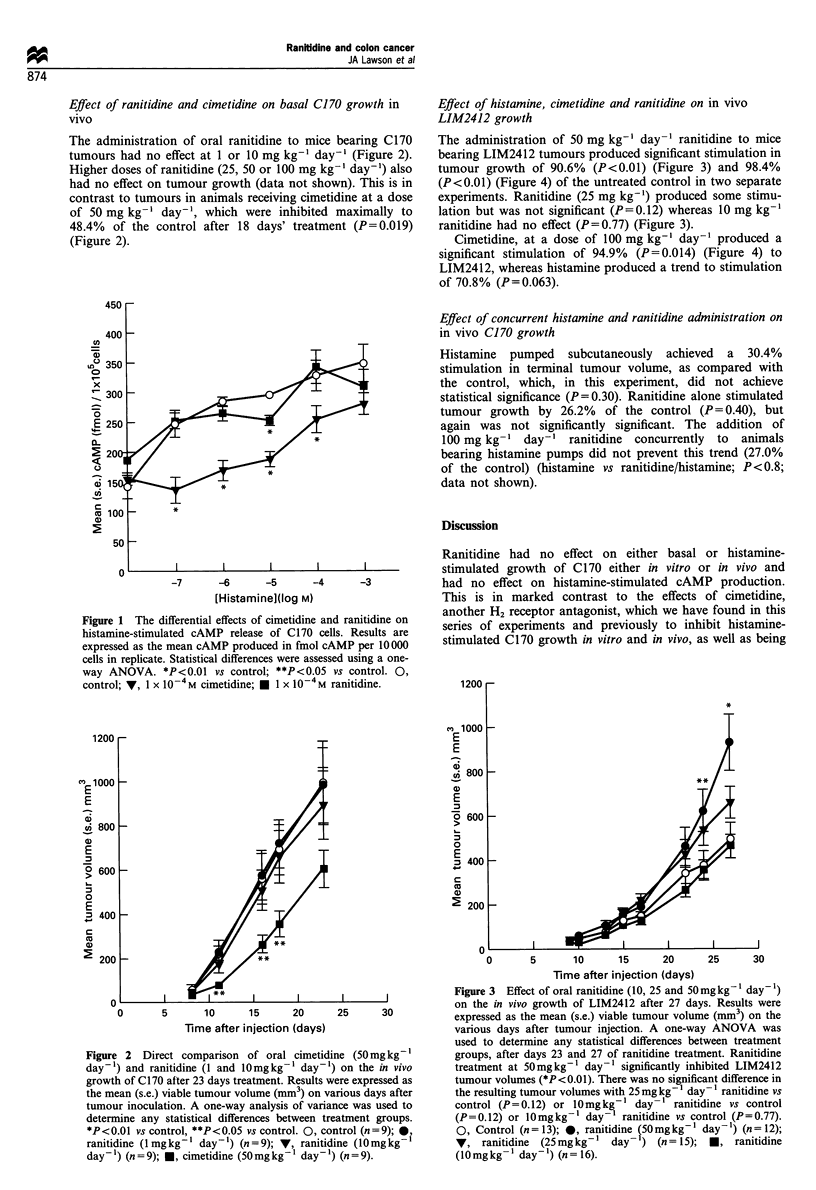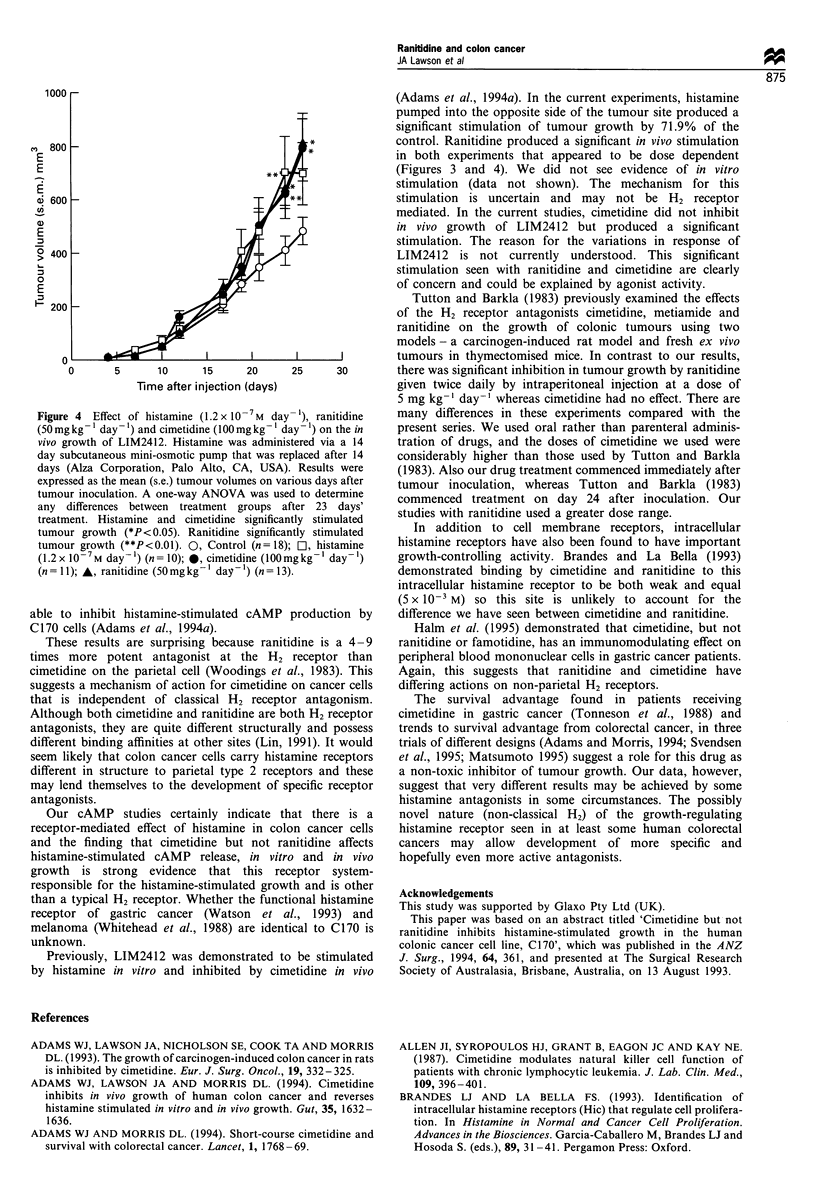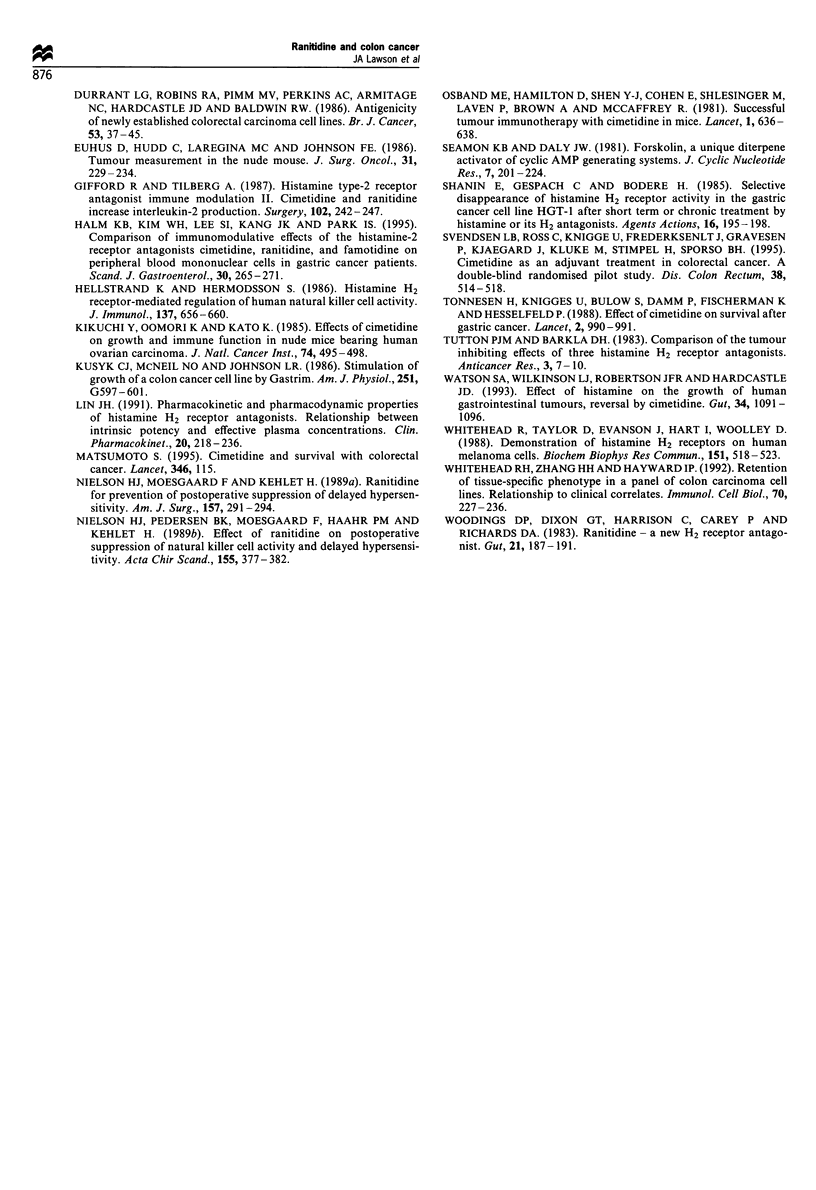# Ranitidine and cimetidine differ in their in vitro and in vivo effects on human colonic cancer growth.

**DOI:** 10.1038/bjc.1996.155

**Published:** 1996-04

**Authors:** J. A. Lawson, W. J. Adams, D. L. Morris

**Affiliations:** UNSW Department of Surgery, The St. George Hospital, Kogarah Sydney, Australia.

## Abstract

Histamine has recently been shown to be a growth factor for some gastric and colorectal cancer cells. Previous studies have shown that cimetidine blocks in vitro and in vivo histamine-stimulated growth and cAMP release from the human colonic cancer cell line, C170. In this study, ranitidine, another H2 receptor antagonist, did not affect either basal or histamine-stimulated in vitro proliferation of C170, and failed to prevent cAMP release in vitro. Ranitidine did not inhibit in vivo growth of C170 at a dose of 1, 10, 25, 50 or 100 mg/kg, in contrast to 50 mg/kg/day cimetidine, which produced 39.3% inhibition of tumour volume (p<0.01) after 23 days' treatment. Ranitidine did not inhibit in vivo histamine-stimulated growth of C170 cells . LIM2412, another colonic cancer cell line, was significantly stimulated by both cimetidine and ranitidine in vivo. Ranitidine had no effect on in vitro cell proliferation.


British Journal of Cancer (1996) 73, 872-876

© 1996 Stockton Press All rights reserved 0007-0920/96 $12.00

### Ranitidine and cimetidine differ in their *in vitro* and *in vivo* effects on human colonic cancer growth

JA Lawson, WJ Adams and DL Morris

*UNSW Department of Surgery, The St. George Hospital, Kogarah Sydney, N.S.W. 2217 Australia.*

**Summary** Histamine has recently been shown to be a growth factor for some gastric and colorectal cancer cells. Previous studies have shown that cimetidine blocks *in vitro* and *in vivo* histamine-stimulated growth and cAMP release from the human colonic cancer cell line, C170. In this study, ranitidine, another $H_2$ receptor antagonist, did not affect either basal or histamine-stimulated *in vitro* proliferation of C170, and failed to prevent cAMP release *in vitro*. Ranitidine did not inhibit *in vivo* growth of C170 at a dose of 1, 10, 25, 50 or 100 mg $kg^{-1}$, in contrast to 50 mg $kg^{-1}$ $day^{-1}$ cimetidine, which produced 39.3% inhibition of tumour volume ($P<0.01$) after 23 days' treatment. Ranitidine did not inhibit *in vivo* histamine-stimulated growth of C170 cells. LIM2412, another colonic cancer cell line, was significantly stimulated by both cimetidine and ranitidine *in vivo*. Ranitidine had no effect on *in vitro* cell proliferation.

**Keywords:** cimetidine; colon cancer; histamine; ranitidine

We recently reported that histamine is a growth factor for some colorectal cancer cell lines (Adams *et al.*, 1994*a*). The histamine receptor antagonist, cimetidine, has been found to significantly slow the growth of experimentally induced gastrointestinal cancers (Adams *et al.*, 1993, 1994; Watson *et al.*, 1993) and improve survival in patients with gastrointestinal malignancies (Tonnesen *et al.*, 1988; Adams and Morris, 1994; Matsumotos, 1995). Whether this is due to the inhibitory effect of cimetidine on suppressor T-lymphocyte activity (Osband *et al.*, 1981), its stimulation of natural killer (NK) cell activity (Hellstrand and Hermodsson, 1986; Allen *et al.*, 1987; Kikuchi *et al.*, 1985), its stimulation of interleukin 2 production in helper T cells (Gifford and Tilberg, 1987) or its blocking of the direct mitogenic effect of histamine on colon cancer (Adams *et al.*, 1994), is unknown.

Ranitidine is a more potent and clinically well-tolerated histamine $H_2$ receptor antagonist than cimetidine. There has been some conflict in the literature as to whether ranitidine and cimetidine have similar effects on the immune system (Nielson *et al.*, 1989*a*, *b*; Halm *et al.*, 1995).

The effects of ranitidine on cancer growth are not well investigated. The aim of this paper was to examine the effect of ranitidine on the growth of colon cancer, and its effect on the histamine-sensitive human colorectal cancer cell lines, C170 and LIM2412.

### Method

### Cell lines

C170 cells are an adherent cell line (Durrant *et al.*, 1986), which were derived from a patient with a Dukes' C colonic adenocarcinoma (CRC Laboratories, Nottingham, UK). LIM2412 cells are a suspension cell line with some adherent cells present (Whitehead *et al.*, 1992). This cell line was derived from a patient diagnosed with a poorly differentiated colonic adenocarcinoma (Ludwig Institute, Melbourne, Australia). Both these cell lines were grown in RPMI-1640 with 10% fetal calf serum (FCS) under 5% carbon dioxide and refed twice weekly.

### In vitro *cell proliferation assay*

Cells were resuspended in serum containing RPMI-1640 at a concentration of $1\times10^4$ cells 0.2 $ml^{-1}$ and incubated overnight in a 96-well microtitre plate. The supernatant was then removed and replaced with 0.6 $\mu$mol of thymidine (Sigma, St Louis, MO, USA) in serum-free media. After 24 h the supernatant was removed and replaced with histamine (Sigma) and/or ranitidine hydrochloride (Glaxo, Greenford, UK) in serum-free media with untreated controls. Ranitidine was added in replicates of at least three, over a concentration range of $1\times10^{-9}$ M to $1\times10^{-6}$ M. Histamine was added to the cells with/without ranitidine at a concentration range of $1\times10^{-9}$ M to $1\times10^{-7}$ M as $1\times10^{-8}$ M most frequently achieved maximal stimulation (Adams *et al.*, 1994). Each experiment was repeated three times. As a direct measure of DNA replication (Kusyk *et al.*, 1986), 0.1 $\mu$Ci of methyl-[$^3$H]-thymidine (DuPont, NEN, Boston, MA, USA) was added to the wells and incubated for a further 8, 24 and 48 h. The cells were then harvested using a cell harvester (PHD cell harvester, Cambridge Technology, USA) and counted using a beta-counter (Minaxi Tri-carb 4000 series, United Technologies Packard, USA).

### *Statistical analysis*

Results were calculated as a mean percentage of the control (s.e.). Any statistical differences were calculated using a one-way analysis of variance (ANOVA). A *P*-value of less than 0.05 was considered significant.

### *Quantification of intracellular cyclic adenosine monophosphate (cAMP)*

Intracellular cAMP was measured using a monoclonal antibody-based kit (Amersham, UK). C170 cells were harvested and resuspended in serum-free RPMI-1640 with 0.5 mM isobutyl methylxanthine (IBMX, Sigma) at $1.25\times10^5$ cells 0.25 $ml^{-1}$, and incubated in polypropylene tubes at 37°C for 10 min. Histamine aliquots of 0.125 ml were added at a concentration range of $1\times10^{-7}$ to $1\times10^{-3}$ M, with or without the addition of ranitidine at $1\times10^{-4}$ M. Forskolin, a direct stimulator of adenylate cyclase (Seamon and Daly, 1981), was added in triplicate at a final concentration of $1\times10^{-6}$ M. Following the addition of histamine, the cells were incubated for 10 min at 37°C (Shanin *et al.*, 1985), then centrifuged for 3 min at 2000 r.p.m. The supernatant was removed and the cells fixed in 0.5 ml of 0.001 M hydrochloric acid-ethanol chilled at 4°C, to allow for cAMP extraction. After thorough mixing, the tubes were further centrifuged for 15 min at 15 000 r.p.m. Supernanant (0.4 ml) was removed and placed into fresh tubes and the contents dried using a Speed Vac Concentrator (model SVC100H, Savant Instruments, NY, USA) at 60°C for 60 min. These were then

Correspondence: DL Morris
Received 7 August 1995; revised 27 October 1995; accepted 20 November 1995

reconstitued in 2 ml of buffer provided in the kit and cAMP measured.

Results were expressed as fmol of cAMP $1\times10^{-5}$ cells. Each drug concentration was measured in triplicate and the results expressed as a mean (s.e.).

*Nude mouse model*

All animal procedures were carried out under the approval of the University of New South Wales, Australia, Animal Care and Ethics Committee.

*Effect of ranitidine on* in vivo *basal C170 or LIM2412 tumour growth*

C170 or LIM2412 cells were injected subcutaneously into 6–9 week male *nu/nu* mice (Ansto, Lucas Heights, Australia) at a concentration of $1\times10^{6}$ cells 0.1 $ml^{-1}$ RPMI-1640 with 10% FCS. Each treatment group had ten animals per group unless otherwise stated, based on our previous studies, which showed 50 mg $kg^{-1}$ $day^{-1}$ cimetidine to inhibit C170 tumour growth by 44% of the control (Adams *et al.*, 1994).

Animals with C170 tumours received 0, 0.1, 0.2, 0.4 mg $ml^{-1}$ ranitidine hydrochloride in the drinking water, in two separate experiments, which produced an approximate daily intake of 25, 50 or 100 mg $kg^{-1}$ $day^{-1}$ of ranitidine hydrochloride, commencing immediately following tumour injection. Animals inoculated with LIM2412 cells were randomised to receive ranitidine 10, 25 and 50 mg $kg^{-1}$ given in the drinking water. These calculations are based on the observations in our laboratory that the mice drink an average of 5 (s.e. 0.6) ml $day^{-1}$ (Adams *et al.*, 1994). The water bottles containing ranitidine hydrochloride were replaced every 2 days.

Tumour areas were measured twice weekly using vernier callipers. Tumour volumes were calculated using the formula 0.5 length $\times$ $(width)^2$ (Euhus *et al.*, 1986). The mice were sacrificed after a maximum of 28 days after inoculation.

*Effect of concurrent histamine and ranitidine administration on C170*

C170 cells were injected subcutaneously, as above. The animals were than randomly allocated to receive either control (phosphate-buffered saline; PBS) or histamine ($1\times10^{-6}$ M $min^{-1}$) via a 14 day subcutaneous Alzet mini-osmotic pump (Alza Corporation, Palo Alto, CA, USA). Both the control and histamine-treated group received 0.4 mg $ml^{-1}$ (approximately 100 mg $kg^{-1}$ $day^{-1}$) of ranitidine hydrochloride in the drinking water. Treatment continued for 22 days after injection. Mini-osmotic pumps were replaced after 14 days.

*Effect of histamine, cimetidine and ranitidine on* in vivo *LIM2412 growth*

LIM2412 cells were injected as above. The control group received water *ad libitum*. Ranitidine and cimetidine was administered via the drinking water at a dose of 50 and 100 mg $kg^{-1}$ $day^{-1}$ respectively. Histamine was administered via a mini-osmotic pump at a dose of $1.2\times10^{-7}$ M $day^{-1}$ on the opposite flank to the tumour site. Treatment continued for 25 days after tumour inoculation.

*Direct comparison of ranitidine and cimetidine administration on C170*

C170 xenografts were established as above and animals randomised to cimetidine 50 mg $kg^{-1}$ $day^{-1}$, ranitidine 1 or 10 mg $kg^{-1}$ $day^{-1}$ in the drinking water, or untreated control.

*Statistical analysis*

A one-way analysis of variance (ANOVA) was used to measure the significant differences as a result of the different treatment regimens in all experiments. Only animals with actively growing tumours were used as part of the statistical analysis.

### Results

In vitro*: C170*

Ranitidine had no effect on basal growth in five experiments in which histamine produced a stimulation in cell proliferation, of which three were significant ($P<0.05$) to a maximum of 142.8% of control at $1\times10^{-8}$ M histamine (Table I). Ranitidine, at a concentration of $1\times10^{-8}$ to $1\times10^{-6}$ M, failed to inhibit the histamine-stimulated cell proliferation. Histamine did not stimulate cell proliferation in assays shorter than 48 h (data not shown).

*LIM2412*

Neither histamine nor ranitidine affected basal growth of LIM2412 cells. Because of our inability to show a significant *in vitro* stimulation with histamine we were unable to study the effect of ranitidine on histamine-stimulated *in vitro* growth (data not shown).

*Quantification of intracellular cAMP, C170*

The addition of histamine alone to C170 cells significantly stimulated cAMP production in a dose-dependent manner. This effect was antagonised by cimetidine (Figure 1). In contrast, ranitidine did not inhibit histamine-stimulated cAMP release.

**Table I** Effect of ranitidine and histamine on *in vitro* cell proliferation of C170 cells after 48 h with a tritiated thymidine label

| *Ranitidine* | *Histamine* | *1* | *2* | *3* | *4* | *5* |
|---|---|---|---|---|---|---|
| 0 | – | 100.0 (2.3) | 100.0 (4.4) | 100.0 (3.8) | 100.0 (12.9) | 100.0 (4.2) |
| $1\times10^{-8}$ M | – | – | – | 119.1 (8.4)* | – | – |
| $1\times10^{-7}$ M | – | – | 98.1 (8.3) | 121.1 (4.0)* | – | – |
| $1\times10^{-6}$ M | – | 106.5 (4.2) | – | – | 114.0 (13.4) | 106.2 (6.5) |
| – | $1\times10^{-9}$ M | 95.6 (7.2) | 125.3 (11.0)* | – | 114.3 (13.4) | 115.1 (7.7) |
| – | $1\times10^{-8}$ M | 142.8 (21.0)* | 132.5 (7.5)* | 117.3 (4.3)* | 126.2 (14.6) | 119.9 (7.7) |
| – | $1\times10^{-7}$ M | 141.8 (23.0)* | 106.1 (9.6) | – | 126.9 (14.6) | 112.1 (12.4) |
| $1\times10^{-8}$ M | $1\times10^{-8}$ M | – | – | 114.2 (6.2)* | – | – |
| $1\times10^{-7}$ M | $1\times10^{-8}$ M | – | 128.4 (8.5)* | 124.4 (9.1) | – | – |
| $1\times10^{-6}$ M | $1\times10^{-8}$ M | 121.8 (10.0) | – | – | 116.1 (16.1) | 121.6 (8.2) |

Results are expressed as a mean per cent (s.e.m.) of the control of each experiment ($n=5$). Results of five separate experiments are presented. *$P<0.05$ *vs* control using a one-way ANOVA.

*Effect of ranitidine and cimetidine on basal C170 growth* in vivo

The administration of oral ranitidine to mice bearing C170 tumours had no effect at 1 or 10 mg $kg^{-1}$ $day^{-1}$ (Figure 2). Higher doses of ranitidine (25, 50 or 100 mg $kg^{-1}$ $day^{-1}$) also had no effect on tumour growth (data not shown). This is in contrast to tumours in animals receiving cimetidine at a dose of 50 mg $kg^{-1}$ $day^{-1}$, which were inhibited maximally to 48.4% of the control after 18 days' treatment ($P$=0.019) (Figure 2).

**Figure 1** The differential effects of cimetidine and ranitidine on histamine-stimulated cAMP release of C170 cells. Results are expressed as the mean cAMP produced in fmol cAMP per 10 000 cells in replicate. Statistical differences were assessed using a one-way ANOVA. *$P$<0.01 *vs* control; **$P$<0.05 *vs* control. ○, control; ▼, $1\times10^{-4}$ M cimetidine; ■ $1\times10^{-4}$ M ranitidine.

**Figure 2** Direct comparison of oral cimetidine (50 mg $kg^{-1}$ $day^{-1}$) and ranitidine (1 and 10 mg $kg^{-1}$ $day^{-1}$) on the *in vivo* growth of C170 after 23 days treatment. Results were expressed as the mean (s.e.) viable tumour volume ($mm^3$) on various days after tumour inoculation. A one-way analysis of variance was used to determine any statistical differences between treatment groups. *$P$<0.01 *vs* control, **$P$<0.05 *vs* control. ○, control ($n$=9); ●, ranitidine (1 mg $kg^{-1}$ $day^{-1}$) ($n$=9); ▼, ranitidine (10 mg $kg^{-1}$ $day^{-1}$) ($n$=9); ■, cimetidine (50 mg $kg^{-1}$ $day^{-1}$) ($n$=9).

*Effect of histamine, cimetidine and ranitidine on* in vivo *LIM2412 growth*

The administration of 50 mg $kg^{-1}$ $day^{-1}$ ranitidine to mice bearing LIM2412 tumours produced significant stimulation in tumour growth of 90.6% ($P$<0.01) (Figure 3) and 98.4% ($P$<0.01) (Figure 4) of the untreated control in two separate experiments. Ranitidine (25 mg $kg^{-1}$) produced some stimulation but was not significant ($P$=0.12) whereas 10 mg $kg^{-1}$ ranitidine had no effect ($P$=0.77) (Figure 3).

Cimetidine, at a dose of 100 mg $kg^{-1}$ $day^{-1}$ produced a significant stimulation of 94.9% ($P$=0.014) (Figure 4) to LIM2412, whereas histamine produced a trend to stimulation of 70.8% ($P$=0.063).

*Effect of concurrent histamine and ranitidine administration on* in vivo *C170 growth*

Histamine pumped subcutaneously achieved a 30.4% stimulation in terminal tumour volume, as compared with the control, which, in this experiment, did not achieve statistical significance ($P$=0.30). Ranitidine alone stimulated tumour growth by 26.2% of the control ($P$=0.40), but again was not significantly significant. The addition of 100 mg $kg^{-1}$ $day^{-1}$ ranitidine concurrently to animals bearing histamine pumps did not prevent this trend (27.0% of the control) (histamine *vs* ranitidine/histamine; $P$<0.8; data not shown).

### Discussion

Ranitidine had no effect on either basal or histamine-stimulated growth of C170 either *in vitro* or *in vivo* and had no effect on histamine-stimulated cAMP production. This is in marked contrast to the effects of cimetidine, another $H_2$ receptor antagonist, which we have found in this series of experiments and previously to inhibit histamine-stimulated C170 growth *in vitro* and *in vivo*, as well as being

**Figure 3** Effect of oral ranitidine (10, 25 and 50 mg $kg^{-1}$ $day^{-1}$) on the *in vivo* growth of LIM2412 after 27 days. Results were expressed as the mean (s.e.) viable tumour volume ($mm^3$) on the various days after tumour injection. A one-way ANOVA was used to determine any statistical differences between treatment groups, after days 23 and 27 of ranitidine treatment. Ranitidine treatment at 50 mg $kg^{-1}$ $day^{-1}$ significantly inhibited LIM2412 tumour volumes (*$P$<0.01). There was no significant difference in the resulting tumour volumes with 25 mg $kg^{-1}$ $day^{-1}$ ranitidine *vs* control ($P$=0.12) or 10 mg $kg^{-1}$ $day^{-1}$ ranitidine *vs* control ($P$=0.12) or 10 mg $kg^{-1}$ $day^{-1}$ ranitidine *vs* control ($P$=0.77). ○, Control ($n$=13); ●, ranitidine (50 mg $kg^{-1}$ $day^{-1}$) ($n$=12); ▼, ranitidine (25 mg $kg^{-1}$ $day^{-1}$) ($n$=15); ■, ranitidine (10 mg $kg^{-1}$ $day^{-1}$) ($n$=16).

**Figure 4** Effect of histamine ($1.2 \times 10^{-7}$ M day$^{-1}$), ranitidine (50 mg kg$^{-1}$ day$^{-1}$) and cimetidine (100 mg kg$^{-1}$ day$^{-1}$) on the *in vivo* growth of LIM2412. Histamine was administered via a 14 day subcutaneous mini-osmotic pump that was replaced after 14 days (Alza Corporation, Palo Alto, CA, USA). Results were expressed as the mean (s.e.) tumour volumes on various days after tumour inoculation. A one-way ANOVA was used to determine any differences between treatment groups after 23 days' treatment. Histamine and cimetidine significantly stimulated tumour growth (*$P<0.05$). Ranitidine significantly stimulated tumour growth (**$P<0.01$). ○, Control ($n=18$); □, histamine ($1.2 \times 10^{-7}$ M day$^{-1}$) ($n=10$); ●, cimetidine (100 mg kg$^{-1}$ day$^{-1}$) ($n=11$); ▲, ranitidine (50 mg kg$^{-1}$ day$^{-1}$) ($n=13$).

able to inhibit histamine-stimulated cAMP production by C170 cells (Adams *et al.*, 1994*a*).

These results are surprising because ranitidine is a 4–9 times more potent antagonist at the $H_2$ receptor than cimetidine on the parietal cell (Woodings *et al.*, 1983). This suggests a mechanism of action for cimetidine on cancer cells that is independent of classical $H_2$ receptor antagonism. Although both cimetidine and ranitidine are both $H_2$ receptor antagonists, they are quite different structurally and possess different binding affinities at other sites (Lin, 1991). It would seem likely that colon cancer cells carry histamine receptors different in structure to parietal type 2 receptors and these may lend themselves to the development of specific receptor antagonists.

Our cAMP studies certainly indicate that there is a receptor-mediated effect of histamine in colon cancer cells and the finding that cimetidine but not ranitidine affects histamine-stimulated cAMP release, *in vitro* and *in vivo* growth is strong evidence that this receptor system-responsible for the histamine-stimulated growth and is other than a typical $H_2$ receptor. Whether the functional histamine receptor of gastric cancer (Watson *et al.*, 1993) and melanoma (Whitehead *et al.*, 1988) are identical to C170 is unknown.

Previously, LIM2412 was demonstrated to be stimulated by histamine *in vitro* and inhibited by cimetidine *in vivo* (Adams *et al.*, 1994*a*). In the current experiments, histamine pumped into the opposite side of the tumour site produced a significant stimulation of tumour growth by 71.9% of the control. Ranitidine produced a significant *in vivo* stimulation in both experiments that appeared to be dose dependent (Figures 3 and 4). We did not see evidence of *in vitro* stimulation (data not shown). The mechanism for this stimulation is uncertain and may not be $H_2$ receptor mediated. In the current studies, cimetidine did not inhibit *in vivo* growth of LIM2412 but produced a significant stimulation. The reason for the variations in response of LIM2412 is not currently understood. This significant stimulation seen with ranitidine and cimetidine are clearly of concern and could be explained by agonist activity.

Tutton and Barkla (1983) previously examined the effects of the $H_2$ receptor antagonists cimetidine, metiamide and ranitidine on the growth of colonic tumours using two models – a carcinogen-induced rat model and fresh *ex vivo* tumours in thymectomised mice. In contrast to our results, there was significant inhibition in tumour growth by ranitidine given twice daily by intraperitoneal injection at a dose of 5 mg kg$^{-1}$ day$^{-1}$ whereas cimetidine had no effect. There are many differences in these experiments compared with the present series. We used oral rather than parenteral administration of drugs, and the doses of cimetidine we used were considerably higher than those used by Tutton and Barkla (1983). Also our drug treatment commenced immediately after tumour inoculation, whereas Tutton and Barkla (1983) commenced treatment on day 24 after inoculation. Our studies with ranitidine used a greater dose range.

In addition to cell membrane receptors, intracellular histamine receptors have also been found to have important growth-controlling activity. Brandes and La Bella (1993) demonstrated binding by cimetidine and ranitidine to this intracellular histamine receptor to be both weak and equal ($5 \times 10^{-3}$ M) so this site is unlikely to account for the difference we have seen between cimetidine and ranitidine.

Halm *et al.* (1995) demonstrated that cimetidine, but not ranitidine or famotidine, has an immunomodulating effect on peripheral blood mononuclear cells in gastric cancer patients. Again, this suggests that ranitidine and cimetidine have differing actions on non-parietal $H_2$ receptors.

The survival advantage found in patients receiving cimetidine in gastric cancer (Tonneson *et al.*, 1988) and trends to survival advantage from colorectal cancer, in three trials of different designs (Adams and Morris, 1994; Svendsen *et al.*, 1995; Matsumoto 1995) suggest a role for this drug as a non-toxic inhibitor of tumour growth. Our data, however, suggest that very different results may be achieved by some histamine antagonists in some circumstances. The possibly novel nature (non-classical $H_2$) of the growth-regulating histamine receptor seen in at least some human colorectal cancers may allow development of more specific and hopefully even more active antagonists.

**Acknowledgements**
This study was supported by Glaxo Pty Ltd (UK).
This paper was based on an abstract titled 'Cimetidine but not ranitidine inhibits histamine-stimulated growth in the human colonic cancer cell line, C170', which was published in the *ANZ J. Surg.*, 1994, **64**, 361, and presented at The Surgical Research Society of Australasia, Brisbane, Australia, on 13 August 1993.

### References

ADAMS WJ, LAWSON JA, NICHOLSON SE, COOK TA AND MORRIS DL. (1993). The growth of carcinogen-induced colon cancer in rats is inhibited by cimetidine. *Eur. J. Surg. Oncol.*, **19**, 332–325.

ADAMS WJ, LAWSON JA AND MORRIS DL. (1994). Cimetidine inhibits *in vivo* growth of human colon cancer and reverses histamine stimulated *in vitro* and *in vivo* growth. *Gut*, **35**, 1632–1636.

ADAMS WJ AND MORRIS DL. (1994). Short-course cimetidine and survival with colorectal cancer. *Lancet*, **1**, 1768–69.

ALLEN JI, SYROPOULOS HJ, GRANT B, EAGON JC AND KAY NE. (1987). Cimetidine modulates natural killer cell function of patients with chronic lymphocytic leukemia. *J. Lab. Clin. Med.*, **109**, 396–401.

BRANDES LJ AND LA BELLA FS. (1993). Identification of intracellular histamine receptors (Hic) that regulate cell proliferation. In *Histamine in Normal and Cancer Cell Proliferation. Advances in the Biosciences.* Garcia-Caballero M, Brandes LJ and Hosoda S. (eds.), **89**, 31–41. Pergamon Press: Oxford.

DURRANT LG, ROBINS RA, PIMM MV, PERKINS AC, ARMITAGE NC, HARDCASTLE JD AND BALDWIN RW. (1986). Antigenicity of newly established colorectal carcinoma cell lines. *Br. J. Cancer*, **53**, 37–45.

EUHUS D, HUDD C, LAREGINA MC AND JOHNSON FE. (1986). Tumour measurement in the nude mouse. *J. Surg. Oncol.*, **31**, 229–234.

GIFFORD R AND TILBERG A. (1987). Histamine type-2 receptor antagonist immune modulation II. Cimetidine and ranitidine increase interleukin-2 production. *Surgery*, **102**, 242–247.

HALM KB, KIM WH, LEE SI, KANG JK AND PARK IS. (1995). Comparison of immunomodulative effects of the histamine-2 receptor antagonists cimetidine, ranitidine, and famotidine on peripheral blood mononuclear cells in gastric cancer patients. *Scand. J. Gastroenterol.*, **30**, 265–271.

HELLSTRAND K AND HERMODSSON S. (1986). Histamine $H_2$ receptor-mediated regulation of human natural killer cell activity. *J. Immunol.*, **137**, 656–660.

KIKUCHI Y, OOMORI K AND KATO K. (1985). Effects of cimetidine on growth and immune function in nude mice bearing human ovarian carcinoma. *J. Natl. Cancer Inst.*, **74**, 495–498.

KUSYK CJ, MCNEIL NO AND JOHNSON LR. (1986). Stimulation of growth of a colon cancer cell line by Gastrim. *Am. J. Physiol.*, **251**, G597–601.

LIN JH. (1991). Pharmacokinetic and pharmacodynamic properties of histamine $H_2$ receptor antagonists. Relationship between intrinsic potency and effective plasma concentrations. *Clin. Pharmacokinet.*, **20**, 218–236.

MATSUMOTO S. (1995). Cimetidine and survival with colorectal cancer. *Lancet*, **346**, 115.

NIELSON HJ, MOESGAARD F AND KEHLET H. (1989*a*). Ranitidine for prevention of postoperative suppression of delayed hypersensitivity. *Am. J. Surg.*, **157**, 291–294.

NIELSON HJ, PEDERSEN BK, MOESGAARD F, HAAHR PM AND KEHLET H. (1989*b*). Effect of ranitidine on postoperative suppression of natural killer cell activity and delayed hypersensitivity. *Acta Chir Scand.*, **155**, 377–382.

OSBAND ME, HAMILTON D, SHEN Y-J, COHEN E, SHLESINGER M, LAVEN P, BROWN A AND MCCAFFREY R. (1981). Successful tumour immunotherapy with cimetidine in mice. *Lancet*, **1**, 636–638.

SEAMON KB AND DALY JW. (1981). Forskolin, a unique diterpene activator of cyclic AMP generating systems. *J. Cyclic Nucleotide Res.*, **7**, 201–224.

SHANIN E, GESPACH C AND BODERE H. (1985). Selective disappearance of histamine $H_2$ receptor activity in the gastric cancer cell line HGT-1 after short term or chronic treatment by histamine or its $H_2$ antagonists. *Agents Actions*, **16**, 195–198.

SVENDSEN LB, ROSS C, KNIGGE U, FREDERKSENLT J, GRAVESEN P, KJAEGARD J, KLUKE M, STIMPEL H, SPORSO BH. (1995). Cimetidine as an adjuvant treatment in colorectal cancer. A double-blind randomised pilot study. *Dis. Colon Rectum*, **38**, 514–518.

TONNESEN H, KNIGGES U, BULOW S, DAMM P, FISCHERMAN K AND HESSELFELD P. (1988). Effect of cimetidine on survival after gastric cancer. *Lancet*, **2**, 990–991.

TUTTON PJM AND BARKLA DH. (1983). Comparison of the tumour inhibiting effects of three histamine $H_2$ receptor antagonists. *Anticancer Res.*, **3**, 7–10.

WATSON SA, WILKINSON LJ, ROBERTSON JFR AND HARDCASTLE JD. (1993). Effect of histamine on the growth of human gastrointestinal tumours, reversal by cimetidine. *Gut*, **34**, 1091–1096.

WHITEHEAD R, TAYLOR D, EVANSON J, HART I, WOOLLEY D. (1988). Demonstration of histamine $H_2$ receptors on human melanoma cells. *Biochem Biophys Res Commun.*, **151**, 518–523.

WHITEHEAD RH, ZHANG HH AND HAYWARD IP. (1992). Retention of tissue-specific phenotype in a panel of colon carcinoma cell lines. Relationship to clinical correlates. *Immunol. Cell Biol.*, **70**, 227–236.

WOODINGS DP, DIXON GT, HARRISON C, CAREY P AND RICHARDS DA. (1983). Ranitidine – a new $H_2$ receptor antagonist. *Gut*, **21**, 187–191.